# Biomedical engineer density per hospital bed: A medical device quality indicator for Mexican Healthcare System

**DOI:** 10.1371/journal.pone.0350988

**Published:** 2026-06-09

**Authors:** Luis G. Ayala, Nadezhda Aguilar Blas, Norma Patricia Navor Galeana

**Affiliations:** 1 Área de Diseño e Innovación Tecnológica en Equipo Médico, Departamento de Ingeniería Biomédica, Instituto Nacional de Ciencias Médicas y Nutrición Salvador Zubirán, Ciudad de México. México; 2 Área de Diseño e Innovación Tecnológica en Equipo Médico, Departamento de Ingeniería Biomédica, Instituto Nacional de Ciencias Médicas y Nutrición Salvador Zubirán, Ciudad de México, México; 3 Departamento de Evaluación Tecnológica, Instituto Nacional de Rehabilitación Luis Guillermo Ibarra Ibarra, Ciudad de México, México; Institute of Public Health from Guanajuato State, MEXICO

## Abstract

**Background:**

The measurement of health indicators that enable the estimation of a system’s capacity to provide adequate patient care constitutes a highly relevant factor in the evaluation of healthcare systems. Traditionally, these indicators include variables such as physician density, nursing staff, and other human resources; however, a critical component that often receives less attention is the capacity of healthcare institutions to sustain high-quality infrastructure and medical technology, which are essential to ensuring the continuity, safety, and efficiency of healthcare services. In this context, an important complementary metric would be the density of biomedical engineers within hospital settings, as these professionals are required to design, evaluate, regulate, acquire, maintain, and manage medical technologies and healthcare infrastructure. Current WHO metrics measure biomedical engineer density per 10,000 inhabitants, not reflecting their direct relationship with medical devices and hospital infrastructure.

**Objective:**

To develop a novel biomedical engineer density indicator per hospital beds (*ρBEhb*) that relates workforce capacity to medical devices rather than general population, and to evaluate its applicability within the Mexican healthcare system.

**Methods:**

The *ρBEhb* calculates the ratio between biomedical engineers providing professional services (internal or mixed staff) and available hospital beds for a given year. The methodological framework follows PAHO/WHO ‘Health Indicators’ guidelines. Data from the Mexican healthcare system (2017–2022) were analyzed alongside international benchmarks.

**Results:**

The Mexican healthcare system showed generally positive *ρBEhb* trends from 2017 to 2022, with a −0.53% decrease (2017−2018) and a notable 14.41% increase (2020−2021) coinciding with the SARS-CoV-2 pandemic. While Mexico's absolute number of biomedical engineers is comparable to the USA, *ρBEhb* analysis reveals a dual structural deficit — insufficient hospital beds and inadequate biomedical engineering workforce — when adjusted for population size and international standards.

**Conclusions:**

The *ρBEhb* indicator exposes Mexico's dual deficit in hospital beds and biomedical engineers relative to population needs. This metric provides a more relevant measure for assessing medical device management capacity and healthcare quality than traditional population-based indicators.

## Introduction

Medical devices are essential components of modern healthcare systems, playing a critical role in diagnosis, treatment, and patient monitoring [1]. The effective management, maintenance, and safe operation of these technologies require specialized biomedical engineering professionals [2,3]. However, current metrics for assessing biomedical engineering workforce adequacy remain disconnected from the actual objects of their professional intervention.

The World Health Organization (WHO) measures biomedical engineer density using a population-based indicator: the number of biomedical engineers per 10,000 inhabitants. This metric is justified because trained biomedical engineering professionals are required to design, evaluate, regulate, acquire, maintain, and manage medical technologies, and should be considered part of the health workforce.

However, this population-based approach fails to account for the direct relationship between biomedical engineers and their primary work domain—medical devices within hospital settings. This limitation becomes particularly evident when comparing healthcare systems with different levels of hospital infrastructure development, where population-based metrics may obscure critical workforce gaps in device management capacity [3].

As of May 13, 2026, a search was conducted on lens.org using the keywords ‘Biomedical engineer’ and ‘Indicator’ within the Title, Abstract, Keywords, and Field of Study categories, yielding 22 academic works. A review of the themes and contents of these records revealed that only the 2024 work by Ayala addresses the creation of an indicator for biomedical engineers. This specific work, authored by one of the contributors to the present study, was previously uploaded to the medRxiv repository as a direct precedent to the research presented herein [4]. **The** ***ρBEhb*** **represents the first such metric proposed in the literature**.

Hospital beds serve as a proxy indicator for medical device density, as each bed typically requires multiple devices for patient care, monitoring, and life support, and they are measured as beds maintained, staffed, and immediately available for use in hospitals. Consequently, the ratio between biomedical engineers and hospital beds offers a more functionally relevant measure of workforce capacity to manage medical technology infrastructure. This approach aligns with the fundamental principle that biomedical engineers’ workload is primarily determined by the volume and complexity of medical equipment rather than by population size alone [5,6].

Previous studies have documented significant disparities in healthcare infrastructure across countries and regions. Mexico, with a population of 131 million, faces particular challenges: healthcare coverage reaches only 77% compared to over 89% in other OECD countries, health spending represents just 5.7% of GDP (far below the OECD average of 9.2%), and the health system has comparatively few hospital beds, physicians, and nurses with substantial geographical disparities between urban and rural areas. Understanding these dynamics is crucial for health policy planning, resource allocation, and ultimately, for ensuring patient safety through adequate medical device management [7,8].

This study addresses that gap by introducing the biomedical engineer density indicator per hospital beds (*ρBEhb*), a metric specifically designed to reflect the functional relationship between biomedical engineering professionals and the medical device ecosystem they manage, offering a more operationally relevant measure than existing population-based approaches. We apply this indicator to analyze the Mexican healthcare system from 2017 to 2022, providing insights into workforce trends during a period that includes the unprecedented challenges of the SARS-CoV-2 pandemic. The methodological framework follows PAHO/WHO guidelines for health indicator development from the Strategic Plan 2020–2025, ensuring international comparability and methodological rigor [8].

The objectives of this study are to develop and validate the *ρBEhb* indicator — a novel contribution to health workforce metrics — based on established health indicator methodology, to analyze trends in biomedical engineer density in the Mexican healthcare system over a six-year period, and to compare the results with international benchmarks to identify workforce gaps and infrastructure deficits.

## Materials and methods

The *ρBEhb* calculates the ratio between biomedical engineers providing professional services (internal or mixed staff) and available hospital beds for a given year. The methodological framework follows PAHO/WHO ‘Health Indicators’ guidelines. Data from the Mexican healthcare system (2017–2022) were analyzed alongside international benchmarks.

### Conceptual Framework and Indicator Justification

Current WHO metrics measure biomedical engineer density using a population-based indicator (*ρBE* × 10,000), calculated as the ratio of certified biomedical engineers (*BE*) to the population (*P*) in a specific geographic area and time, multiplied by 10,000 [9]:


$$\begin{array}{c} \;\rho BE=\frac{BE}{P}\times \text{1}\text{0},\text{0}\text{0}\text{0}\; \end{array}$$ (1)

The WHO justifies this indicator by stating that global health systems need biomedical engineering professionals and technicians and they should be considered part of the health workforce [2]. This approach follows methodologies established for other healthcare professionals such as physicians and nurses, who maintain direct contact with individuals requiring medical care.

However, a fundamental distinction exists in the professional intervention object between these professions. While physicians and nurses provide direct diagnosis, care, treatment, and rehabilitation to patients, biomedical engineers intervention focuses on medical devices (MDs) that healthcare professionals use to perform clinical activities. Without these devices, comprehensive healthcare delivery would not be possible

This disparity becomes particularly evident when considering care level distribution. Approximately 90% of essential actions for universal health coverage can be performed in primary healthcare units [5], where the focus is on prevention, treatment, and management of conditions not requiring hospitalization. Consequently, diagnostic, treatment, and rehabilitation medical devices are more prevalent in higher care levels where hospitalization and management of complex conditions, such as chronic metabolic diseases, occur in specialty hospitals.

Given that biomedical engineers’ workload is primarily determined by the volume and complexity of medical equipment rather than population size, a population-based indicator fails to accurately reflect workforce adequacy relative to medical technology infrastructure. Therefore, a novel approach is needed that correctly relates healthcare professionals to their professional intervention object.

### Hospital beds as proxy for medical device density

The challenge in directly measuring medical device density lies in the large number and variety of MDs across healthcare systems. To address this, we propose using hospital beds (*HB*) as a standardized proxy indicator for medical device infrastructure.

A hospital bed is defined as a bed installed in hospitals and rehabilitation centers to accommodate patients during observation, diagnosis, care, or treatment [10]. These beds are used to measure indicators such as hospital discharges, occupancy, and length of stay, covering beds for the treatment of both acute and chronic diseases. Hospital beds are considered the functional units of specialty medical hospitals and represent areas where medical device concentration is highest.

The hospital beds per 1,000 inhabitants (*HB* × 1,000) indicator is well-established, continuously updated, and widely reported by health systems at national, regional, and city levels through organizations such as the World Bank and WHO, making data readily accessible for calculations [5,10]. The indicator is calculated as:


$$\begin{array}{c} \rho HB=\frac{HB}{P}\times \text{1}\text{0}\text{0}\text{0}\; \end{array}$$ (2)

where *HB* represents the number of hospital beds in a health system (HS) at a specific time and location, and *P* is the population in that period.

### Mathematical development of the *ρBEhb* indicator

The proposed biomedical engineer density per hospital beds (*ρBEhb*) indicator establishes a direct relationship between biomedical engineering workforce and hospital infrastructure:


$$\begin{array}{c} \rho BEhb=\frac{\frac{\rho BE}{\text{1}\text{0}}}{\rho HB}\; \end{array}$$ (3)

where *BE* represents the number of biomedical engineers and *HB* represents hospital beds in a health system for a specified time and geographic location.

To calculate this indicator using available normalized data, a mathematical derivation is required. The WHO reports biomedical engineer density per 10,000 inhabitants (*BE* × 10,000), expressed as:


$$\begin{array}{c} BE\times \text{1}\text{0},\text{0}\text{0}\text{0}=\frac{BE}{P}\times \text{1}\text{0},\text{0}\text{0}\text{0} \end{array}$$ (4)

For comparability with the *HB* × 1,000 indicator, the *BE* × 10,000 value must be adjusted by dividing by 10 to obtain biomedical engineers per 1,000 inhabitants:


$$\begin{array}{c} BE\times \text{1},\text{0}\text{0}\text{0}=\frac{BE\times \;b\text{1}\text{0},\text{0}\text{0}\text{0}}{\text{1}\text{0}} \end{array}$$ (5)

This adjustment is unnecessary if both indicators originally use the same multiplicative scale. The complete calculation of *ρBEhb* using normalized data is developed as follows:


$$\begin{array}{c} \rho BEhb=\frac{BE}{HB}=\;\frac{BE\times \text{1}\text{0},\text{0}\text{0}\text{0}}{HB\times \text{1}\text{0},\text{0}\text{0}\text{0}}=\frac{BE\times \text{1},\text{0}\text{0}\text{0}}{HB\times \text{1},\text{0}\text{0}\text{0}}=\frac{\frac{BE\times \text{1}\text{0},\text{0}\text{0}\text{0}}{\text{1}\text{0}}}{HB\times \text{1}\text{0}\text{0}\text{0}} \end{array}$$ (6)

The proposed mathematical formulation demonstrates that when relating the normalized values, the population variable (P) becomes non-relevant for calculating the indicator, focusing solely on biomedical engineers and their professional intervention object (hospital beds as proxy for medical devices).

### Methodological reference framework

The development of the *ρBEhb* indicator follows the methodological guidelines established by the Pan American Health Organization (PAHO) and WHO for health indicator construction, as outlined in the PAHO Strategic Plan 2020–2025 and the Health Indicators conceptual framework [11]. This approach ensures:

- Conceptual validity: the indicator measures what it intends to measure
- Reliability: consistency in measurement across time and contexts
- Comparability: standardized calculation allowing cross-system comparisons
- Data accessibility: utilization of routinely collected health system data
- Policy relevance: actionable information for workforce planning and resource allocation

### Study population and inclusion criteria

This study focuses specifically on biomedical engineers (*BE*s) providing professional services within hospital settings. Following WHO classification [2], we include *BE*s working under two organizational models:

- Internal staff model: Biomedical engineers employed directly by the hospital as part of the institutional workforce
- Mixed staff model: Biomedical engineers working through combined internal employment and external service contracts

The study excludes biomedical engineers working exclusively in:

- Research and development institutions
- Medical device sales and procurement companies
- Regulatory agencies
- Educational institutions
- Exclusive external service provision without hospital affiliation

This delimitation ensures the indicator reflects workforce capacity directly involved in medical device management, maintenance, and quality assurance within healthcare delivery settings.

### Data sources and collection

a) *Biomedical Engineer Data*

Data on the number of biomedical engineers was obtained from two primary sources:

- WHO Global Health Workforce Statistics and the Medical Devices unit reports [2,9] National health system employment records and workforce databases
- For the Mexican case study, data were extracted from:
- Ministry of Health (Secretaría de Salud) official employment statistics
- National Health System annual reports (2017–2022) [12]
- National Institute of Statistics and Geography [13]
- Mexican Social Security Institute (IMSS) workforce registries [14]
- Institute for Social Security and Services for State Workers (ISSSTE) personnel records [15]

b) *Hospital Bed Data*

Hospital bed data were obtained from:

- World Bank Health Nutrition and Population Statistics database [10]
- OECD Health Statistics [5,6]
- WHO Global Health Observatory data repository [2]
- National health system infrastructure reports [16–18]
- For Mexico, specific sources included:
- Ministry of Health Statistical Yearbooks (Anuarios Estadísticos)
- National Health Information System
- Hospital infrastructure registries by state and institution

c) *Temporal and Geographic Scope*

- Study period: 2017–2022 (six-year longitudinal analysis)
- Geographic scope: Mexican healthcare system (national level)
- Data frequency: Annual measurements
- Reference date: December 31st of each year for workforce counts; annual averages for hospital bed availability

d) *Indicator Calculation and Analysis*
  i) Annual *ρBEhb* Calculation

For each year from 2017 to 2022, the *ρBEhb* indicator was calculated following equation (6):

- Extraction of *BE* × 10,000 data from national workforce records
- Conversion to *BE*×1,000 by dividing by 10 (equation 5)
- Extraction of *HB* × 1,000 data from infrastructure databases
- Division of *BE* × 1,000 by HB × 1,000 to obtain *ρBEhb*

This analysis identified:

- Overall trend direction (positive or negative)
- Peak variations and inflection points
- Relationship with external events (e.g., SARS-CoV-2 pandemic)
- International Benchmarking

Mexican *ρBEhb* values were compared with available international reference data from:

- High-income OECD countries
- Latin American and Caribbean countries with comparable economic development
- WHO regional benchmarks

Comparisons considered both absolute *ρBEhb* values and population-adjusted metrics to contextualize Mexico's position relative to international standards.

### Data quality and indicator robustness considerations

The reliability and validity of the *ρBEhb* indicator are contingent upon the quality of underlying health system data. Several factors influence indicator robustness: Comprehensive national legislation recognizing medical devices as essential healthcare infrastructure. Systematic and standardized data collection procedures for workforce and infrastructure metrics. Regular auditing and validation of reported data. Clear definitions and consistent application of inclusion criteria for biomedical engineers and hospital beds and centralized health information systems with interoperability across institutions.

Limiting factors affecting indicator reliability: Incomplete workforce registries, particularly for mixed staff models. Inconsistent reporting across different healthcare subsystems (public, social security, private). Variations in hospital bed definitions and counting methodologies. Geographic disparities in data collection rigor and Temporal delays in data reporting and validation.

For the Mexican case, data quality varies across institutions and regions. The federal Ministry of Health maintains the most comprehensive databases, while state-level and private sector data may have gaps. These limitations are acknowledged and discussed in the context of results interpretation.

In summary, Table 1 presents the technical specifications for the 𝝆*BEhb* developed for this indicator, based on information provided by PAHO/WHO in 2018.

**Table 1:** Technical Specification for the Density of Biomedical Engineers per Hospital Beds *(ρBEhb*).

Indicator Title	Density of Biomedical Engineers per Hospital Beds (*ρBEhb*)
**Definition of the Indicator**	This indicator calculates the relationship between the number of biomedical engineers (*BE*s) and the hospital beds (HBs) available in a healthcare system (HS) for a specific year.
**Purpose of the Indicator**	The development of this indicator provides a reference value for making comparisons between the density of biomedical engineers per hospital beds (*ρBEhb*) in a healthcare system for a specific year. With this data, stakeholders and decision-makers can compare the system’s temporal progression and make suggestions or interventions. It offers a way to compare *ρBEhb* broadly rather than specifically.
**Interpretation**	The obtained ratio can be compared over time against itself and among other densities obtained in national healthcare systems considered robust and efficient. Similarly, such comparisons can be made with local systems within the same national territory to identify regions, states, or areas with advancements and deficiencies. This comparison and the ability to calculate the missing number of BEs and HBs in a healthcare system (HS) to be comparable with other HSs allow for identifying the appropriate number of engineers needed to manage, purchase, repair, and maintain medical equipment within an HS.
**Uses**	Comparison of biomedical engineering services in a broad and non-specific manner. This can include comparisons between countries, regions, or within the same healthcare system (HS) across different years.
**Calculation Method**	Number of biomedical engineers divided by the hospital beds in a healthcare system (HS) reported for a specific year.
**Type of Indicator**	Healthcare Services Indicator. It is a ratio, as it involves two measures that are not conceptually related.
**Unit of Measurement**	Biomedical Engineers / Hospital Beds (BE/HB)
**Measurement Frequency**	Annual
**Reference Area**	National or local.
**Reference Period**	Annual
**Source of Data**	WHO, World Bank, and reports from national and institutional sources on employment, health, and statistics.
**Limitations**	This index is designed to provide a general, broad, and non-specific overview of the density of biomedical engineers in a national or local healthcare system. While it is possible to calculate this ratio for individual hospitals by relating BEs to HBs, the varying profiles of specialized care hospitals mean they will serve different patient profiles and have different equipment. Therefore, the needs of each institution will differ. Comparing healthcare institutions within a HS can be insightful, but the results cannot be directly related due to these differences.
**Technical Notes**	For this calculation, the numerator is the number of biomedical engineers obtained from reports by ministries of health, statistics, or labor. The denominator is the number of hospital beds reported in reports from healthcare facilities or the healthcare system (HS) for a specific year, as provided by the institutions themselves or by ministries of health or statistics. Frequency and quality of the data are crucial for drawing conclusions from this indicator. Indicators such as BEs, hospital beds (HBs), HS, and population (P) are updated at different intervals. For example, some countries do not conduct annual population censuses but do so every 10 years. When making comparisons, it is important to consider the uniformity of data collection across the various mentioned indicators.

Source: Authors’ own elaboration.

This study utilized publicly available, aggregated health system data without individual identifiers. No human subjects were directly involved, and no ethical approval was required. All data sources are cited appropriately, and institutional data sharing agreements were respected.

## Results

Table 2 shows the values of *ρBEhb* calculated using (f) based on the available information from the Mexican Ministry of Health and the population (P) data for Mexico (Mx) from the World Bank. This includes the report on existing hospital beds (HB) in the public healthcare system (HS) and the number of biomedical engineers (BEs) employed. To calculate the indicator, it is necessary to obtain the values of BEx1,000 and HBx1,000 using formulas (c) and (e). Notably, the change in *ρBEhb* in the Mexican HS from 2017 to 2022 has generally been positive, with a negative peak of −0.53% from 2017 to 2018 and an increase of 14.41% from 2020 to 2021, which coincides with the onset of the SARS-CoV-2 pandemic.

**Table 2:** Calculation of the Biomedical Engineers per Hospital Bed Density Indicator (*ρBEhb*) in Mexico for the Period 2017-2022.

	2017	2018	2019	2020	2021	2022
Population in Mexico	122,839,258	124,013,861	125,085,311	125,998,302	126,705,138	127,504,125
Biomedical Engineers (BEs)	369	369	415	457	543	601
Biomedical Engineers per 1,000 inhabitants (BEx1,000)	0.00300	0.00298	0.00332	0.00363	0.00429	0.00471
Hospital Beds (HB)	89,085	89,562	89,538	90,555	94,047	95,780
Hospital Beds per 1,000 (HBx1000)	0.73	0.72	0.72	0.72	0.74	0.75
Density of Biomedical Engineers per Hospital Beds (*ρBEhb*)	0.0041	0.0041	0.0046	0.0050	0.0058	0.0063
Percentage change in *ρBEhb* compared to the previous year	—	−0.53%	12.50%	8.88%	14.41%	8.68%

Source: Prepared by the authors with data from the Mexican Ministry of Health [12] and population data from the World Bank [10].

**Fig 1:**
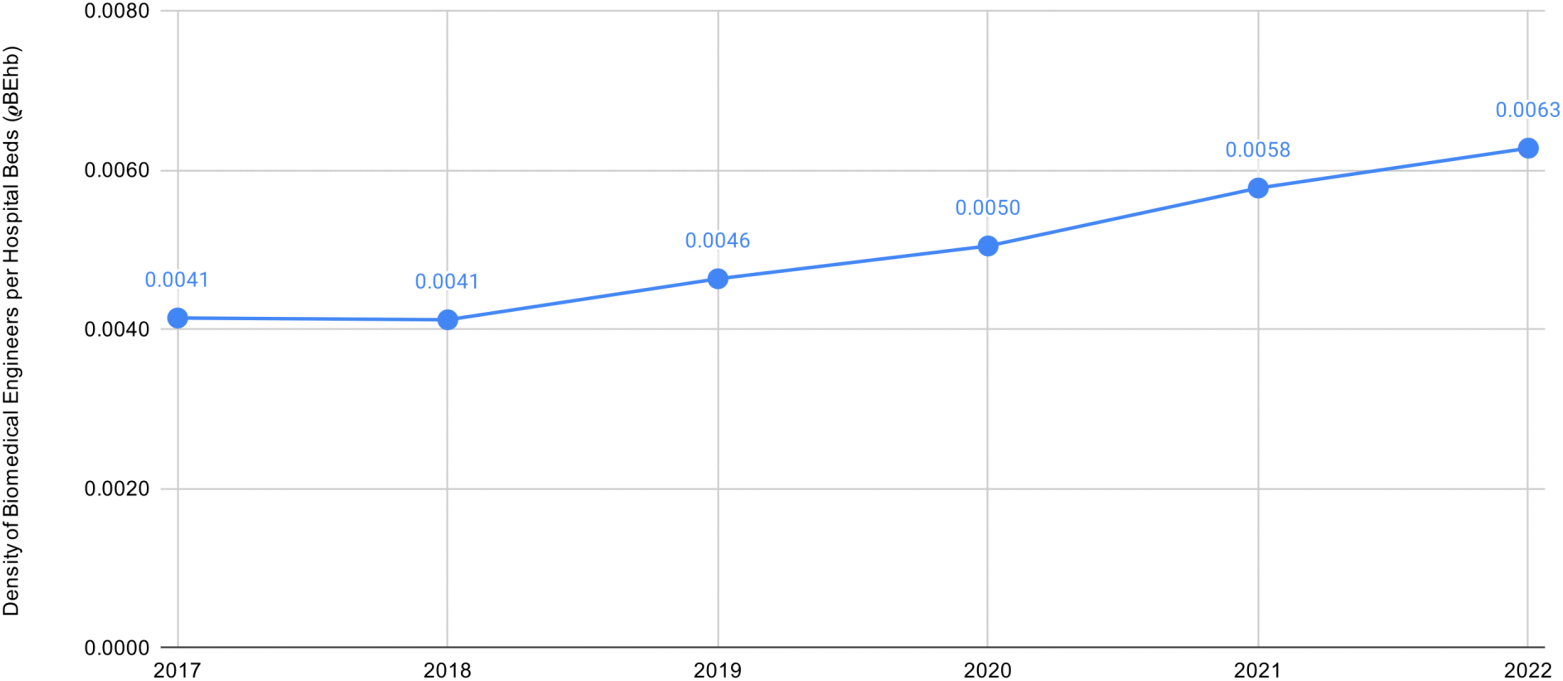
Changes in the Biomedical Engineers per Hospital Bed Density Indicator (*ρBEhb*) in Mexico for the period 2017-2022. Source: Prepared by the authors with data from the Mexican Ministry of Health [12] and population data from the World Bank [10].

The values of *ρBEhb* in Mexico for the period 2017–2022 are shown in Fig 1.

Using data from the WHO for BEs and HB, we have calculated the *ρBEhb* for a selection of countries. The latest reported data for BEs is from 2017, so all data presented will be from that year. Table 3 contains this information and includes the inverse of the *ρBEhb* (*ρBEhb* ⁻ ¹ = 1/*ρBEhb*). The *ρBEhb* can be interpreted as the proportion of BEs per HB; thus, the inverse represents the number of HBs attended to by each BE.

**Table 3:** Calculation of the density of biomedical engineers per hospital beds (*ρBEhb*) for a Selection of Countries in 2017.

Country	*ρBEhb* 2017	1 / *ρBEhb* 2017
China	0.11037	9.1
Denmark	0.03014	33.2
Japan	0.02475	40.4
Mexico	0.02422	41.3
USA	0.02411	41.5
Costa Rica	0.01504	66.5
Spain	0.00741	135.0
Argentina	0.00678	147.6
Brazil	0.00218	459.4

Source: Compiled by the authors with data from [9].

Table 4 contains the biomedical engineer density indicators per 10,000 inhabitants for the same selected countries.

**Table 4:** Density of biomedical engineers per 10,000 inhabitants in 2017.

Country	*ρBEhb* 2017 (WHO)
China	4.79
Japan	3.23
Denmark	0.79
USA	0.68
Argentina	0.34
Mexico	0.24
Spain	0.22
Costa Rica	0.17
Brazil	0.05

Source: Data from [9].

When comparing the values shown by both indicators, we can immediately see the difference. The WHO indicator relates BEs to the number of people in a specific location and time, which allows us to estimate how many BEs would serve each person in that population. After normalizing 10,000, we can determine how many BEs would serve 10,000 individuals. This metric would be relevant if the professional intervention object of BEs were on individuals. Physicians directly care for people, but *BEs* address the needs of MDs. Therefore, the interpretation of the WHO indicator is less relevant because the demand for *BEs* is determined by the health system, not the population. While health systems should be designed to serve a specific population size, in practice, this is not always the case, and they operate according to available resources. Some health systems may be under-resourced and unable to meet the required level of care for their population, while others might have excess resources, potentially providing higher quality care.

The proposed indicator (*ρBEhb*) allows for a clear interpretation of the results. As shown in Table 3, HS can be compared to determine, for example, that a BE in Mexico (Mx) and in the United States of America (USA) are servicing nearly the same number of hospital beds (Mx = 41.3, USA = 41.5). Thus, for the size of the health systems in Mx and USA, the number of BEs is comparable. This interpretation cannot be made using the WHO’s indicator of BEs per 10,000 inhabitants, as it only shows a marked difference between Mx (0.24) and USA (0.68), which is dependent on the population size. The WHO indicator might suggest that Denmark (0.79) has a HS most like the USA in terms of BEs, but this is not necessarily accurate given the different contexts of the health systems.

**Fig 2:**
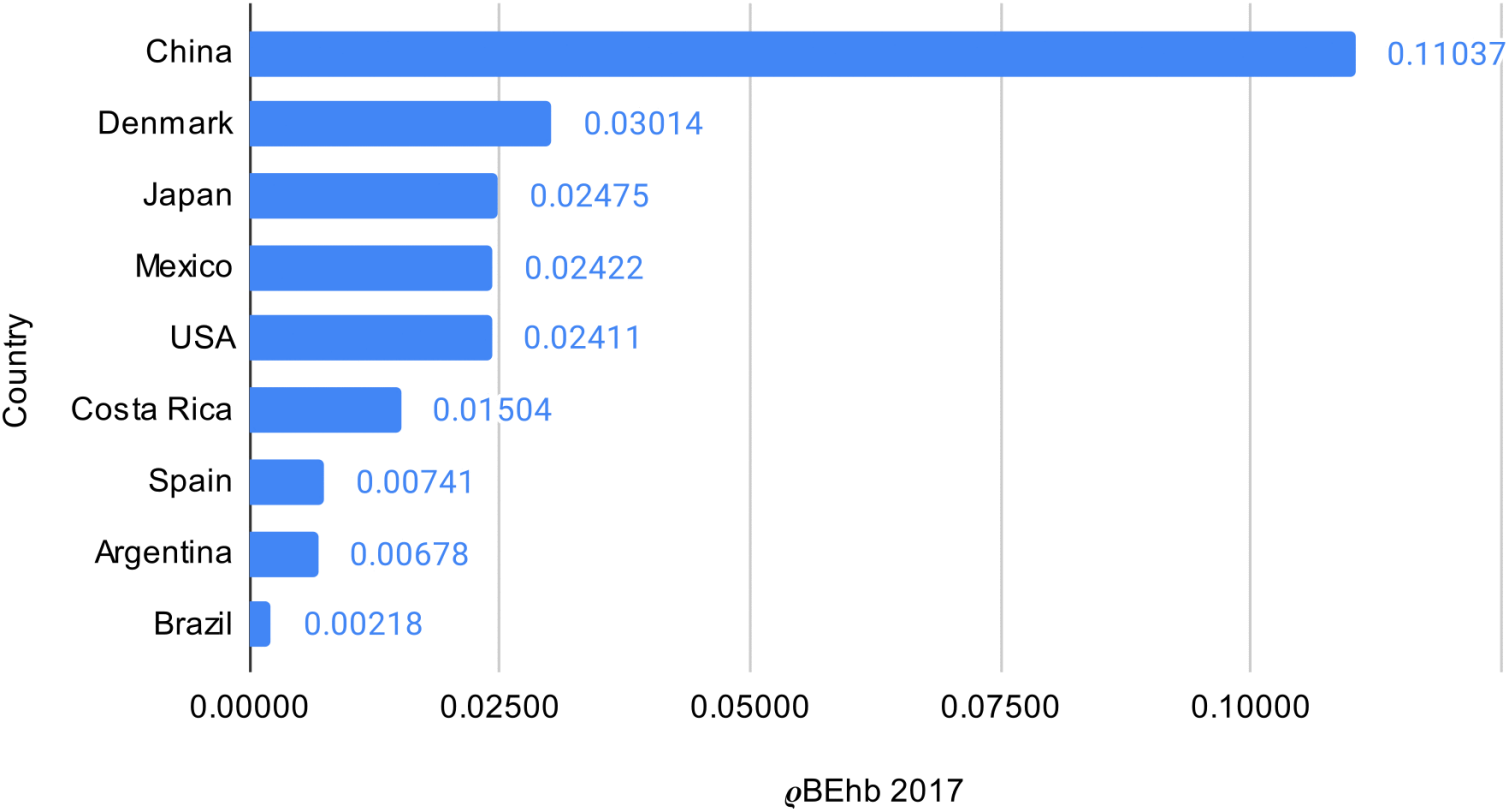
Calculation of density of biomedical engineers per hospital beds (*ρBEhb*) in selected countries for 2017. Source: Prepared by the authors with data from [9].

**Fig 3:**
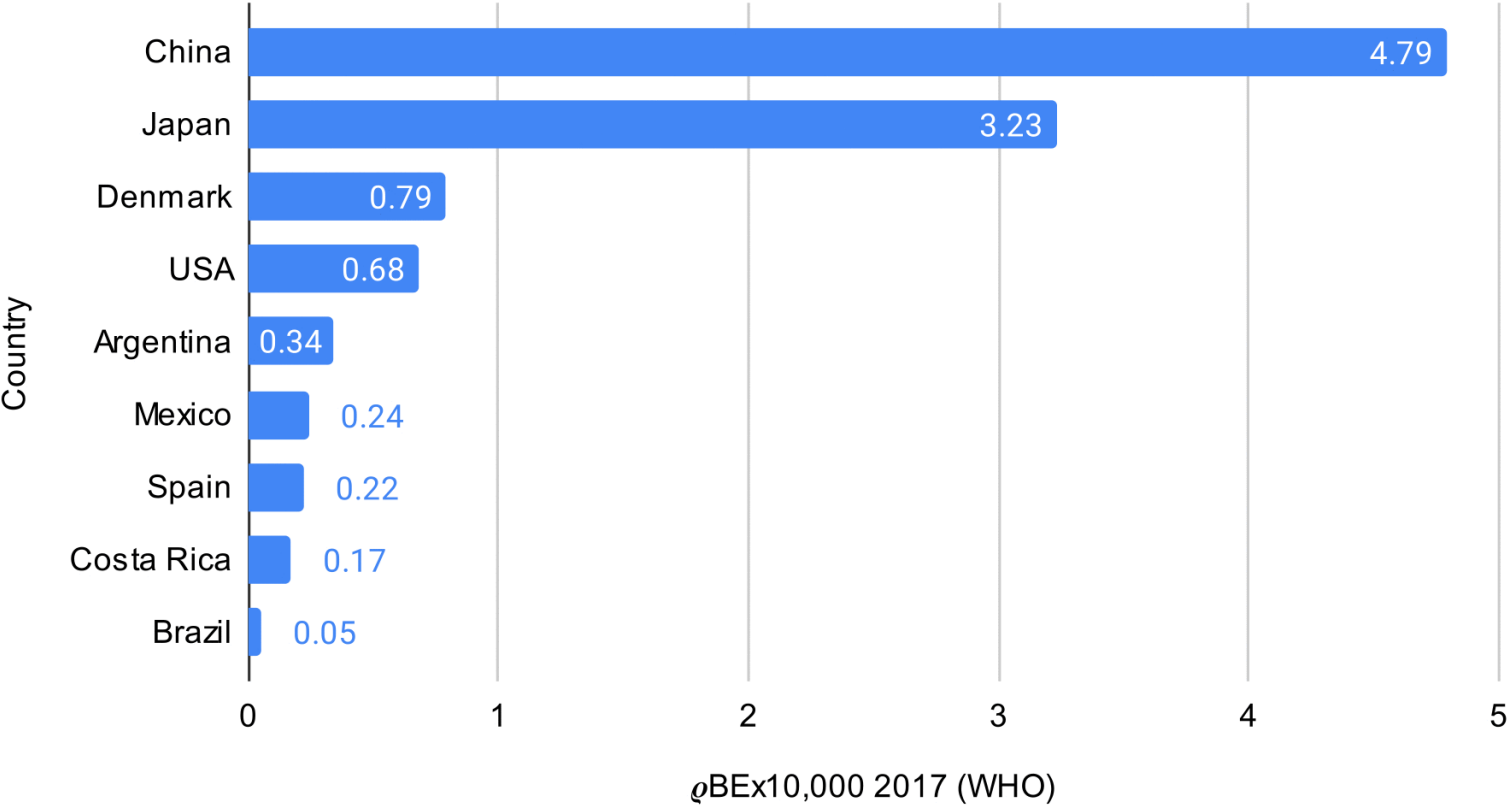
Density of biomedical engineers per 10,000 Inhabitants (𝝆BEx10,000) in selected countries for 2017. Source: Prepared by the authors with data from [9].

In Fig 2 and Fig 3, we visualize the differences between the two indicators.

Another important discussion concerns the adequacy of the number of BEs in a HS. The WHO indicator does not provide insights into this issue. By using the *ρBEhb* indicator, we can assess the relationship between the current HS and a reference HS. For reference, we will use the average number of hospital beds (HB) reported by OECD member countries in their “Health at a Glance 2019” report, as it provides data for the year 2017, which has become our reference year. According to this report, there are 4.7 HBs per 1,000 inhabitants [19]. Compared to Mexico's HB value for the same year, the country would have a deficit of 3.7 HBs per 1,000 inhabitants.

**Fig 4:**
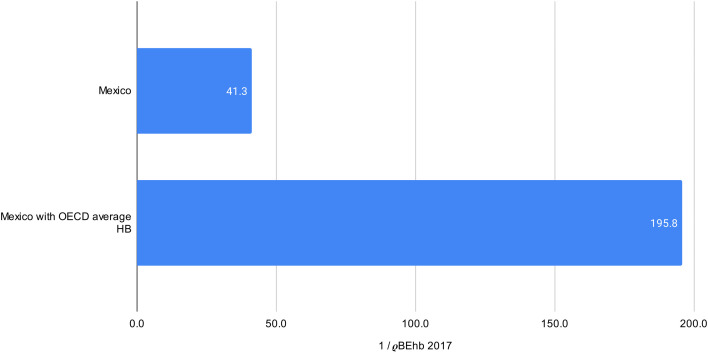
Indicator of biomedical engineers per hospital bed (*ρBEhb*) in Mexico with the values of its healthcare system in 2017 compared to the hospital bed value (4.7 per 1,000 inhabitants) in the OECD average for the same year. Source: Prepared by the authors with data from [9] and [19].

Fig 4 shows the difference in the inverse of *ρBEhb* in Mexico for the year 2017 and compares the value in the healthcare system between the current sector and the reference sector (OECD average).

If the value of *ρBEhb* were to be maintained (the same proportion of BE to HB), it can be calculated that to handle 4.7 hospital beds per 10,000 inhabitants (the OECD average), Mexico would need to increase its BEs by approximately 11,034 (rounded number). Thus, while Mexico had a comparable number of BE to the USA in 2017, to provide care comparable to the OECD average, Mexico would need to address both a deficit in HB and BE.

**Fig 5:**
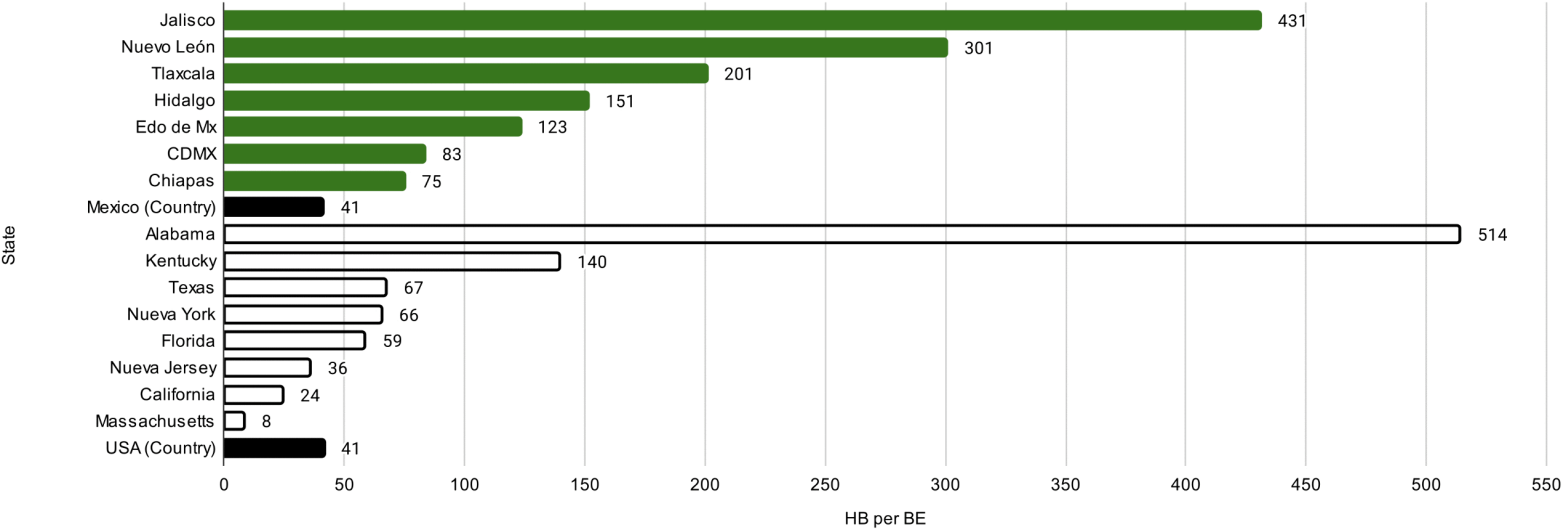
Density of biomedical engineers per hospital bed (*ρBEhb*) in selected states of Mexico and the USA for the year 2022. Source: Created by the authors with data from [12,13,16–18].

The same exercise can be conducted with data from territories and states. Population data can be consulted in censuses, and HB and BE data can be obtained from HS reports. Fig 5 presents the calculation of *ρBEhb* ⁻ ¹ for selected states in the USA and Mexico using 2022 data for hospital beds and biomedical engineers, and 2020 data (the closest year) for population.

**Fig 6:**
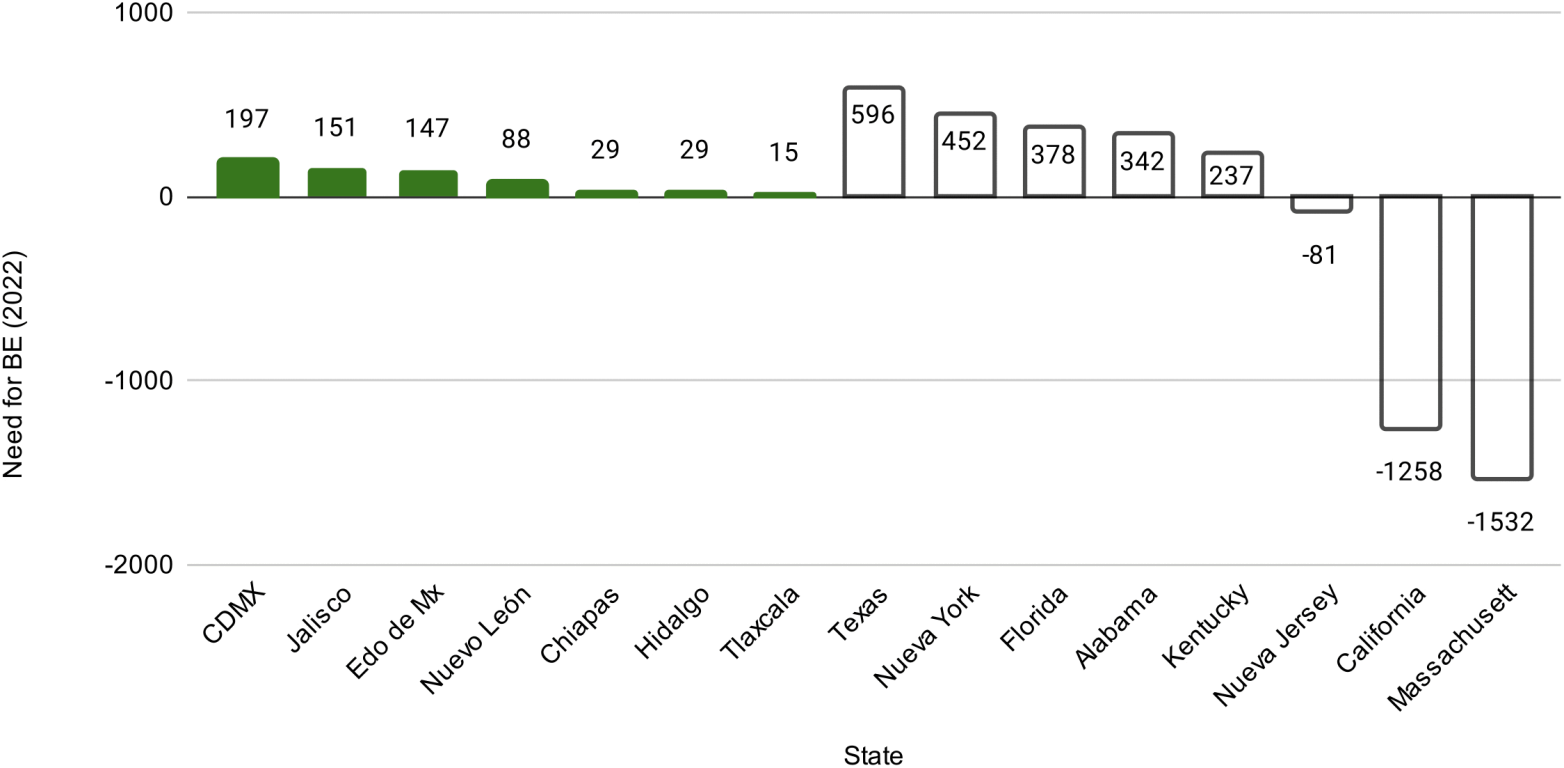
Calculation of the surplus or deficit of biomedical engineers needed in selected states in Mexico and the USA compared to the national *ρBEhb* value. Source: Created by the authors.

With this state-level information, it is possible to calculate the need for or surplus of biomedical engineers in the state health systems, using the national *ρBEhb* value previously calculated and shown in Table 3 as a reference. Fig 6 displays, in positive numbers, the amount of additional biomedical engineers needed, and, in negative numbers, the surplus of engineers required to meet the national value.

Fig 6 shows that, except for California, New Jersey, and Massachusetts, all other state HS in the sampled data exhibit a deficit of BEs. The deficit is more pronounced in the USA state HS compared to those in Mexico, which is a direct consequence of the differences in hospital beds (HB) and overall health system resources.

## Discussion

This study developed the biomedical engineer density per hospital bed (*ρBEhb*) indicator following PAHO/WHO methodological guidelines. Unlike traditional population-based metrics, *ρBEhb* directly relates biomedical engineering workforce to hospital infrastructure, providing a more accurate assessment of medical device management capacity.

### Indicator scope and comparability across heterogeneous healthcare systems

The *ρBEhb* analysis reveals a critical paradox in the Mexican healthcare system. While Mexico's absolute number of biomedical engineers appears comparable to the United States, this masks a fundamental dual deficit: insufficient hospital beds and inadequate biomedical engineering workforce when adjusted for population size and international standards.

Mexico has approximately 1.0 hospital bed per 1,000 inhabitants, significantly below the OECD average of 4.3 beds per 1,000. To reach this benchmark, Mexico would need approximately 430,000 additional hospital beds. Each bed requires multiple medical devices and proportional biomedical engineering support for maintenance, calibration, and safety assurance. Current workforce levels are insufficient for both existing infrastructure and needed expansion.

The *ρBEhb* is a workforce density indicator relative to available infrastructure, analogous to established metrics such as physicians per bed or nurses per bed, none of which distinguish by unit type in their base formulation. Its value does not reside in precisely capturing the technological workload of each institution, but in offering a systemic metric that enables aggregate comparisons across health systems.

Nevertheless, the authors acknowledge that hospital beds are not a homogeneous unit: a bed in an intensive care unit represents a substantially different biomedical engineering workload than a bed in a general ward. Taking this into account, the authors propose as future work the development of an extended version of the indicator incorporating a hospital complexity weighting factor — designated *ρBEhb*-c — operationalized through existing classification frameworks such as the IMSS Hospital Complexity Index, the PAHO three-level care classification, or the Case Mix Index used in U.S. hospital systems as a proxy for technological intensity.

### Public policy strategies for synchronized infrastructure and workforce growth

The *ρBEhb* indicator serves three essential functions: Track workforce adequacy trends within healthcare systems over time. Compare Mexico's capacity against countries with recognized healthcare quality and guide decisions on biomedical engineering education, hiring, and resource allocation

The analysis from 2017 to 2022 showed generally positive trends, with a notable 14.41% increase from 2020 to 2021 during the COVID-19 pandemic. However, this reactive response highlights the need for proactive rather than crisis-driven workforce planning.

The gap identified relative to the OECD average requires a public policy approach operating along two simultaneous and coordinated axes. First, at the normative level, the authors propose that Mexican health authorities adopt the *ρBEhb* as an official planning indicator within the National Health System, linking it directly to hospital infrastructure investment plans. This would ensure that every hospital construction or expansion project carries an explicit biomedical engineering staffing projection, preventing the historical decoupling between available beds and specialized technical personnel.

Second, at the regional level, the authors recommend designing biomedical engineering academic programs and placement positions directed toward states with the greatest *ρBEhb* deficits, articulating universities with local health systems to ensure graduates are absorbed where need is most critical.

Furthermore, given that Mexico's health system encompasses multiple coexisting subsystems — IMSS, ISSSTE, SEDENA, PEMEX, and IMSS-Bienestar — the *ρBEhb* can be disaggregated by institution within each state, identifying not only where deficits exist but in which subsystem they are most critical. This is particularly relevant because hiring strategies and budgetary mechanisms differ substantially across institutions, requiring tailored rather than uniform intervention approaches.

State-level *ρBEhb* analysis should be conducted to identify geographic disparities and guide targeted federal investment in both infrastructure and workforce deployment, ensuring regional equity in healthcare capacity. Implementation of comprehensive tracking systems for medical devices, biomedical engineering workforce distribution, and equipment performance across all healthcare subsystems will provide the data infrastructure necessary for evidence-based decision-making.

These investments represent economic efficiency, as adequate biomedical engineering capacity reduces medical device downtime, prevents premature equipment failure, and improves patient safety—estimated at just 0.5–1% of total health expenditure while yielding substantial returns in service quality and system performance.

### Impact and significance

For Mexico, adopting the *ρBEhb* indicator represents a paradigm shift from abstract population-based metrics to functional capacity assessment. This approach reveals that Mexico's healthcare challenge is not merely workforce shortage but systemic infrastructure underdevelopment requiring simultaneous investment in hospital beds and biomedical engineering professionals.

The indicator's value extends beyond Mexico to other Latin American and middle-income countries facing similar structural challenges. Traditional metrics may suggest adequate workforce levels while actual functional deficits compromise medical technology safety and healthcare quality.

### Study limitations

Data quality varies across Mexican healthcare institutions and subsystems. The fragmentation of Mexico's health system — with subsystems including IMSS, ISSSTE, SEDENA, PEMEX, and IMSS-Bienestar operating independent information platforms — generates inevitable asymmetries in data quality and granularity. This is not exclusive to Mexico: it is a common pattern in health systems of middle-income countries, reinforcing the need to propose generalizable solutions.

To improve biomedical engineering and hospital bed data collection and standardization in Mexico and similar contexts, the authors propose two concrete mechanisms. First, the creation of a National Registry of Biomedical Engineers in Clinical Functions, updated annually and linked to the hospital and medical unit registry, would make the relationship between biomedical professionals and institutions traceable and auditable.

Second, the Mexican Secretariat of Health should incorporate differential budget classifications enabling identification of biomedical engineers by contractual modality — permanent staff, fee-for-service, service contracts, mixed schemes, or research positions. Currently, the absence of this distinction requires indirect estimations that introduce uncertainty into the indicator's numerator.National-level aggregates obscure regional disparities requiring more granular analysis. Future research should establish optimal *ρBEhb* thresholds for different hospital complexity levels and correlate workforce ratios with patient outcomes and medical device safety metrics. The time series analyzed in this study (2017–2022) will be subject to update in subsequent work incorporating post-pandemic data (2023–2026), enabling evaluation of the sustainability of the positive trends identified.

## Conclusions

This study introduces the *ρBEhb* as a health workforce indicator that relates biomedical engineer density to hospital bed availability, offering a functionally relevant alternative to existing population-based metrics. Application of the *ρBEhb* to the Mexican healthcare system (2017–2022) reveals a dual structural deficit: insufficient hospital beds relative to population needs and inadequate biomedical engineering workforce capacity against international benchmarks. The *ρBEhb* indicator provides Mexican policymakers with an analytical framework to make evidence-based decisions synchronizing workforce development with infrastructure growth.

Closing this dual deficit requires coordinated action on two fronts: adopting the *ρBEhb* as an official planning indicator within the National Health System — linking every hospital infrastructure investment to an explicit biomedical engineering staffing projection — and directing targeted academic programs toward the states and subsystems with the greatest documented deficits. At the subnational level, disaggregating the *ρBEhb* by state and institution enables identification of not only where workforce gaps exist but in which subsystem — IMSS, ISSSTE, SEDENA, PEMEX, or IMSS-Bienestar — intervention is most urgent. To strengthen the indicator's reliability, future applications should establish a National Registry of Biomedical Engineers in Clinical Functions, incorporate differential budget classifications distinguishing contractual modalities, and develop a complexity-weighted version — *ρBEhb*-c — accounting for differences in hospital technological profiles.

Only through integrated investment in hospital beds and biomedical engineering capacity can Mexico achieve universal health coverage with quality, safety, and equity. The true measure of success will not be comparing absolute workforce numbers to other countries but ensuring sufficient biomedical engineering capacity to safely manage the medical technologies upon which modern healthcare delivery depends.

## Supporting information

S1 File Calculation of *ρBEhb.* Calculation of biomedical engineer density indicator per hospital beds (*ρBEhb*).
(DOCX)

## References

[1] Evangel Chinyere A, Femi O, Opeoluwa Oluwanifemi A, Jane Osareme O, Tolulope O, Ebere Rosita D. Biomedical engineering advances: A review of innovations in healthcare and patient outcomes. Int J Sci Res Arch. 2024;11(1):870–82. doi: 10.30574/ijsra.2024.11.1.0139

[2] World Health Organization. Human resources for medical devices: The role of biomedical engineers. 2017. https://iris.who.int/handle/10665/255261

[3] World Health Organization. Medical devices: Biomedical engineers density (per 10,000 population). WHO Global Health Observatory. https://www.who.int/data/gho/indicator-metadata-registry/imr-details/4584 2025.

[4] Ayala L. Biomedical engineers density indicator per hospital beds. medRxiv. 2024. doi: 10.1101/2024.09.05.24313063

[5] OECD. Health at a glance 2023: OECD indicators. OECD Publishing. 2023. doi: 10.1787/7a7afb35-en

[6] OECD. OECD health statistics 2025. 2025. https://www.oecd.org/en/data/datasets/oecd-health-statistics.html

[7] Pan American Health Organization. PAHO strategic plan 2020-2025 “Equity at the heart of health” - Compendium of impact indicators. 2020. https://www.paho.org/en/documents/paho-strategic-plan-2020-2025-equity-heart-health-compendium-impact-indicators

[8] Pan American Health Organization. PAHO strategic plan 2020-2025 “Equity at the heart of health” - Compendium of outcome indicators. 2020. https://www.paho.org/en/documents/paho-strategic-plan-2020-2025-equity-heart-health-compendium-outcome-indicators

[9] WHO. Number of biomedical engineers. World Health Organization (WHO). https://www.who.int/data/gho/data/indicators/indicator-details/GHO/number-of-biomedical-engineers 2019. Accessed 2024 August 2.

[10] World Bank. World Bank Open Data. 2011. Retrieved 08 19, 2024, from https://data.worldbank.org/

[11] PAHO/WHO. OPS/OMS | Indicadores de salud: aspectos conceptuales y operativos. Pan American Health Organization. 2018. https://www3.paho.org/hq/index.php?option=com_content&view=article&id=14405:health-indicators-conceptual-and-operational-considerations&Itemid=0&lang=es#gsc.tab=0

[12] Secretaría de Salud. Recursos en Salud Sectorial. http://www.dgis.salud.gob.mx/contenidos/basesdedatos/da_recursos_gobmx.html 2024. Accessed 2024 August 13.

[13] INEGI. Número de habitantes. Cuéntame de México. https://cuentame.inegi.org.mx/poblacion/habitantes.aspx?tema=P 2020. Accessed 2024 August 14.

[14] IMSS. Informe al ejecutivo federal y al congreso de la unión sobre la situación financiera y los riesgos del instituto mexicano del seguro social 2023-2024. Instituto Mexicano del Seguro Social. 2024. https://www.imss.gob.mx/sites/all/statics/pdf/informes/20232024/19-informe-completo.pdf

[15] ISSSTE. Anuario estadístico 2024. issste.gob.mx. 2024. Accessed May 25, 2026. Available from: https://www.issste.gob.mx/datosabiertos/anuarios/anuarios2024.html

[16] KFF. Hospital Beds per 1,000 Population by Ownership Type. https://www.kff.org/other/state-indicator/beds-by-ownership/?currentTimeframe=0&sortModel=%7B%22colId%22:%22Location%22,%22sort%22:%22asc%22%7D#notes 2023. Accessed 2024 August 21.

[17] Census Bureau US. 2020 Census Apportionment Results. https://www.census.gov/data/tables/2020/dec/2020-apportionment-data.html 2021. Accessed 2024 August 21.

[18] U. S. Bureau of Labor Statistics. 17-2031 Bioengineers and Biomedical Engineers. Bureau of Labor Statistics. https://www.bls.gov/oes/2022/may/oes172031.htm 2023. Accessed 2024 August 21.

[19] OECD. Health at a Glance 2019 OECD Indicators. OECD. 2019. doi: 10.1787/4dd50c09-en

