# Supplementary material for: Biomedical engineer density per hospital bed: A medical device quality indicator for Mexican Healthcare System
Source: PLoS One. 2026 Jun 9;21(6):e0350988. doi: 10.1371/journal.pone.0350988 (PMC13249187; doi:10.1371/journal.pone.0350988)
Supplement: S1 File — Calculation of biomedical engineer density indicator per hospital beds (ρBEhb). (DOCX) [file pone.0350988.s001.docx]

Appendix 1

### **Calculation of Biomedical Engineering Density (ρBEhb)**

To perform the calculation, it is necessary to have data for:

- Biomedical Engineers per 1,000 inhabitants (BEx1,000), and
- Hospital beds per 1,000 inhabitants (HBx1,000).

The division is performed using equation (6) to obtain the ρBEhb.

If these values are not available, the calculation can be performed based on:

- Population,
- Hospital beds, and
- Biomedical Engineers.

It must be noted that these data must coincide in both geographical space and time period.

The first step is to obtain HBx1,000 by applying equation (2). Next, calculate BEx1,000 using equation (5). Finally, apply equation (6) using the previously obtained results.

**Calculation example for Mexico City (CDMX)**

As an example, we will calculate the ρBEhb for Mexico City (CDMX) for the year 2022. All healthcare system data have been retrieved from the Mexican Ministry of Health database (Secretaría de Salud, 2024), and population data from INEGI (INEGI, 2020).

- Population: 9,209,944 people
- Hospital beds: 16,103 beds
- Biomedical Engineers: 193 engineers

To obtain HBx1,000, we apply (2):

$HB\times1000=\frac{HB}{P}\times1000 =\frac{16,103}{9,209,944}\times1000=1.748$

Now we will calculate BEx1,000 by applying (4); however, due to the characteristics of the data, instead of multiplying by 10,000, we will multiply by 1,000. Mathematically, this is equivalent to solving (4) and then (5).

$BE\times1,000 =\frac{BE}{P}\times1,000=\frac{193}{9,209,944}\times1,000=0.021$

As a verification of (1), let's perform the same calculation, and we will see that we obtain the same result in (6). First, by substituting in (1):

$\rho BEhb=\frac{BE}{HB}$ = $\frac{193}{16,103}=0.0120$

And now in (6):

$\rho BEhb=\frac{BE}{HB}=\frac{BE\times1,000}{HB\times1,000}=\frac{0.021}{1.748}=0.0120$

This is how the calculation of ρBEhb is performed.

**Calculating the difference in the number of BE between the national and local systems.**

To determine the difference in the number of Biomedical Engineers (BE) between a local and a national healthcare system, the national ρBEhb is multiplied by the local HB (Hospital Beds), and the local BE count is then subtracted from this product. This approach applies a ratio-based normalization using the ρBEhb as the reference benchmark.To illustrate the above, the difference between the number of Biomedical Engineers in Mexico as a country versus the number of Biomedical Engineers in Mexico City (CDMX) will be calculated. First, the ρBEhb for Mexico (ρBEhb_Mx_) must be determined. Using data from the WHO (WHO, 2024) and the World Bank (World Bank, 2011), we obtain the ρBEhb_Mx_ by applying equation (6):

${\rho BEhb}_{Mx}=\frac{{BE}_{Mx}\times1,000}{{HB}_{Mx}\times1,000}=\frac{0.024}{0.99}=0.024$

We then perform the ratio-based normalization using the ρBEhb_Mx_ and the values for HB_CDMX_ and BE_CDMX_.

${\left( {\rho BEhb}_{Mx}\times{BE}_{CDMX}\times1,000 \right)- = b}=\frac{0.024}{0.99}=0.024$

$\left( {\rho BEhb}_{Mx}\times{HB}_{CDMX} \right)-{BE}_{CDMX}=\left( 0.024\times16,103 \right)-193 =197$

Therefore, CDMX has a surplus of 197 BE relative to the national average. If the result of this operation were negative, it would indicate a deficit, as seen in the cases of California and Massachusetts in Figure 6.
